# Cold Side-Effect Effect: Affect Does Not Mediate the Influence of Moral Considerations in Intentionality Judgments

**DOI:** 10.3389/fpsyg.2017.00295

**Published:** 2017-02-28

**Authors:** Rodrigo Díaz, Hugo Viciana, Antoni Gomila

**Affiliations:** ^1^Psychology, Evolution and Cognition (IFISC-CSIC), University of the Balearic IslandsPalma, Spain; ^2^Institute for Philosophy, University of BernBern, Switzerland; ^3^Institute for Advanced Social Studies-Consejo Superior de Investigaciones CientíficasCórdoba, Spain

**Keywords:** side-effect effect, Knobe effect, intentionality, moral, anger, emotion, motivational bias, affective bias

## Abstract

Research has consistently shown that people consider harmful side effects of an action more intentional than helpful side effects. This phenomenon is known as the side-effect effect (SEE), which refers to the influence of moral considerations in judgments of intentionality and other non-moral concepts. There is an ongoing debate about how to explain this asymmetric pattern of judgment and the psychological factors involved in it. It has been posited that affective reactions to agents that bring about harmful side-effects could bias intentionality attributions in these cases, explaining the asymmetric pattern of intentionality judgments that we observe in the SEE. We call this the affective bias hypothesis (ABH). Evidence for the ABH is mixed, with some findings suggesting a role for affective processes, while others suggesting that affective processes play no role in the SEE. A possible explanation for these apparently contradictory results points to affective processes involved in the SEE being confined to anger. In a series of empirical studies, we systematically measured and manipulated participants’ anger in order to test this possibility. Our findings suggest that anger play no role in intentionality judgments in SEE cases, while providing support for a non-emotional motivation to blame as a factor underlying the SEE.

## Introduction

In everyday social life, we constantly attribute mental states such as beliefs and intentions to others. This ability to understand other’s mental states, typically called theory of mind or folk psychology, has been argued to be essential for social functioning and cultural development (Tomasello et al., 2005). Furthermore, moral and legal systems heavily rely on folk psychology. In most cultures, whether or not an action is done intentionally influences judgments of moral wrongness (Barrett et al., 2016). For example, accidentally stepping on someone’s foot is not considered morally wrong, whereas kicking his foot on purpose is completely inappropriate. Most moral and legal systems consider intentionality as a fundamental input for judgment of right or wrong. However, recent investigations suggest that the relationship could also hold in the opposite direction, with moral judgments influencing folk psychological attributions, at least as regards side effects of actions. In a seminal study, Knobe (2003) presented participants with the following vignette:

The vice-president of a company went to the chairman of the board and said, ‘We are thinking of starting a new program. It will help us increase profits, but it will also harm the environment.’
The chairman of the board answered, ‘I don’t care at all about harming the environment. I just want to make as much profit as I can. Let’s start the new program.’
They started the new program. Sure enough, the environment was harmed.

When asked whether or not the chairman of the board intentionally harmed the environment, most participants answered yes (82%). However, when participants were presented with the very same vignette, with the only exception of the word “harm,” which was replaced by “help,” most participants say that the chairman of the board *did not* intentionally help the environment (77%). In both cases the chairman clearly states its indifference to what happens to the environment, and that he only desires to make profit. Consequences for the environment in both cases are thus side effects of the chairman action. Therefore, if one is viewed as intentional the other should also be. But the data shows that most people consider that harming the environment was intentional, whereas helping the environment was not. Inasmuch as the only difference between both vignettes is the moral valence of the side-effect, it is concluded that moral judgments influence intentionality judgments. People usually assign intentionality for negative side effects (Harm cases), but not for positive side effects (Help cases). This phenomenon is known as the side-effect effect (SEE) or the Knobe effect.

One could argue that the abovementioned asymmetry in intentionality judgments is due to some characteristics of the vignette used or the surveyed population. Nevertheless, the effect has been replicated across age groups (Leslie et al., 2006) and cultures (Knobe and Burra, 2006). Further studies used other vignettes involving different protagonists and side effects, and found the same asymmetry between negative and positive side effects (Mele and Cushman, 2007). The asymmetry remains even when participants are presented with both Harm and Help vignettes, and judge the intentionality of both chairmen at the same time (Pinillos et al., 2011). Moreover, the effect has been extended to other concepts, as similar asymmetries due to the influence of moral considerations have been found, for example, with causality and freedom (Knobe, 2010). In conclusion, the SEE has proven to be highly robust.

Since Knobe’s seminar study, a large amount of possible explanations for the SEE have been posited (for a review, see Cova, 2016), to the point that Knobe himself, who defends his own explanatory model for the asymmetry, has acknowledged that probably there is no single explanation for the influence of moral considerations in non-moral judgments (Phillips et al., 2015). Instead, it is to expect that each of the different explanatory factors that have been proposed play some role at least in some cases of the effect.

Among those explanatory factors, it is of special relevance to test factors that could be considered a “biasing” or “distorting” influence. There is an ongoing debate about whether or not the asymmetrical pattern of intentionality attributions seen in SEE cases constitutes an error of judgment or not. Some accounts for the SEE claim that the asymmetry is legitimate. For example, Knobe (2010) claims that moral considerations are part of our competence with the concept of intentional action, and Uttich and Lombrozo (2010) argue that it is rational to take into account moral considerations when attributing mental states to agents because norm-breaking behavior is more informative than norm-abiding behavior. However, other explanations of the SEE argue that the asymmetrical pattern constitutes an error or irrational bias, caused by some “external” factor that interferes with how we should attribute intentionality. Whether or not judgments in SEE cases are legitimate is relevant for philosophical and psychological theorizing, as dominant theories of intentional action (Adams, 1986) and intention attribution (Gopnik and Wellman, 1992) do not take into account moral considerations. If the SEE asymmetry is legitimate, those theories should be revised, but if the asymmetry constitutes an error those theories could remain unaffected.

The most influential explanation of the SEE as an error is the motivational bias account (Nadelhoffer, 2004, 2006; Alicke, 2008; Alicke and Rose, 2012). It posits that a desire to blame the agent in cases of negative side-effects (e.g., the chairman that harms the environment) can bias intentionality attributions. Importantly, they posit a possible psychological mechanism that can trigger this desire to blame: affective processes. This hypothesis has been subject of controversy since the early days of the SEE literature, and arguments and evidence keep accumulating both in favor of and against it. In this paper, we directly test the role of affective processes in the SEE. In the following section, we will explain in more detail the mechanisms by which affective processes could distort intentionality judgments. We will also present the studies that have tested the role of affective processes in the SEE to date, and argue that there is a gap in the empirical literature that has to be filled in order to advance the debate. In the following sections, we present three studies designed in order to fill this gap, the results, and their implications to the debate around how to explain the SEE.

## The Motivational Bias Account and the Role of Affective Processes in the SEE

Thomas Nadelhoffer built on previous research by Alicke (2000) to explain the SEE asymmetry in terms of a motivational bias. According to Nadelhoffer (2004, 2006), the moral blameworthiness of an agent can bias judgments of intentionality regarding his action or its side effects. While normative models describe blame assessments as a sequence in which people evaluate the agent’s mental states and constraints of the situation in order to reach a decision, the psychology of blame is judged to be very different. Due to adaptive pressures to quickly identify wrongdoers, people blame first and search for mitigating circumstances later. These quick blame judgments might then act expansively on intentionality attributions.

According to Mark Alicke’s Culpable Control Model of blame (CCM) (Alicke, 2000), blame attribution depends on the agent freedom to bring about desired outcomes, or control. There are three components of control: mental states, behavior, and consequences. Connections among these components yield three structural links: volitional behavior control (the relationship between mental states and behavior), causal control (the relationship between behavior and consequences) and volitional outcome control (relationship between mental states and consequences). The harder these relationships are, the more control people attribute to the agent, thus more blame is assigned. The main point in CCM is that control judgments are influenced by spontaneous evaluations, which are positive or negative reactions to an agent’s mental states, behavior and/or outcomes. Strong negative reactions make people process information in a “blame validation mode”: their desire to blame makes them construe control evidence in a way that supports the attribution they want to make. In the Harm case of the chairman vignette, negative evaluations of the chairman attitude and the outcome of his action make people attribute intentionality (volitional outcome control) in order to justify blame (Alicke and Rose, 2012).

Despite the fact that most of the literature has assumed that emotion is a necessary component in the motivational bias hypothesis, the biasing effect of spontaneous evaluations on attributions of intentionality is not necessarily emotional. Negative emotions can increase the motivation to blame and thus intentionality ascriptions, but this motivation to blame itself does not depend on emotion. In Mark Alicke’s own words, “spontaneous evaluations are not identical to emotional reactions”; but “Emotions can certainly heighten one’s positive and negative evaluations to information about an event” (Alicke, 2008, p. 184). We must distinguish between the motivational and the affective bias. Affective processes exert their influence increasing the motivation to blame, and thus the affective bias is an extension of the motivational bias and depends on it.

Research on the role of affective processes in judgment and decision making have shown that the abovementioned influence of affective processes can in fact occur, and it has been explained in terms of the information or cognitive content carried by emotions (Clore and Huntsinger, 2007; Lerner et al., 2015). In particular, several research studies showed that emotion can exert an “amplifying” influence in judgments of control and blame. Inducing anger in participants in an unrelated task prior to their judgments led to higher blame attributions (Lerner et al., 1998; Goldberg et al., 1999), perception of events as caused by human agency instead of situational factors (Keltner et al., 1993a), and increased attributions of volitional and causal control (Ask and Pina, 2011). Also consistent with the CCM, increasing anger in mock jurors during a capital punishment trial simulation led to underestimation of mitigating circumstances and increased the probability of assigning a death sentence (Georges et al., 2013).

Nevertheless, the evidence for the role of affective processes in the SEE asymmetry is scarce, indirect, and inconclusive.

On the one hand, Pinillos et al. (2011) found that individuals that scored higher in the Cognitive Reflection Task (CRT) were less likely to show the asymmetric pattern of intentionality judgments involved in the SEE. They interpret their results in terms of system 1 system 2 considerations. To put it simply, as the CRT is related to controlled, system 2 processes, the SEE asymmetry is probably due to system 1, automatic and often emotional processes. Similarly, Cokely and Feltz (2009) found a positive correlation between extraversion and SEE asymmetry width. Since the extraversion personality trait is related to emotional expressiveness, this is also interpreted as evidence in favor of the affective bias hypothesis (ABH). Finally, Ngo et al. (2015) found a relationship between self-reported emotional reactions and intentionality ratings in Harm cases, which was mediated by blame judgments. However, they failed to replicate the correlations between intentionality ratings and individual-difference measures of the previous studies.

On the other hand, Young et al. (2006) found that patients with lesions in the ventromedial prefrontal cortex (VMPFC) of the brain exhibit the same asymmetric pattern of intentionality judgments in the chairman vignette as normal subjects. Given that VMPFC patients show emotional deficits across a wide range of tasks, Young et al. (2006) concluded that emotional processes, or at least those subserved by the VMPFC, do not play a role in the SEE. In support of this conclusion is the fact that this neuropsychological approach has been employed with success to test the role of emotion in moral cognition (Koenigs et al., 2007). Moreover, another clinical population known for its severe emotional deficits, psychopaths, also displays the asymmetry on intentionality judgments that characterizes the SEE (Cardinale et al., 2014).

Some have taken the latter research studies with clinical populations as conclusive evidence against the ABH. However, the emotional deficits of VMPFC patients and psychopaths do not affect anger responses, which are in fact exaggerated in both clinical populations. And as we have seen, anger has been consistently related to blame. Thus, it seems possible that, as normal subjects, VMPFC patients and psychopaths experience anger in response to the SEE scenarios, and this anger motivates a desire to blame that influences their intentionality judgments. In three studies, we measured and manipulated participants’ anger and recorded their responses to SEE cases in order to test this possibility.

## Study 1: Trait Anger

In order to test whether anger responses underlie the asymmetry in intentionality judgments seen in the SEE, we used the Trait Anger Scale (TAS) (Spielberger, 1999) to measure participants’ propensity to feel anger along with their intentionality ratings in the original chairman vignette. Spielberger’s Trait Anger Scale consist on 10 items (e.g., “I am a hotheaded person”) that participants rate in a scale from 1 (“almost never”) to 4 (“almost always”). It has been successfully used in other studies investigating the role of affective processes in judgment and decision making (e.g., Lerner and Keltner, 2000, 2001).

If feeling angry at the events described in the vignettes is what makes people consider negative side-effects as intentional, this judgment would partially depend on people’s tendency to get angry. Thus, if ABH is true, it is to expect that individuals’ propensity to experience anger (as measured by the TAS) will correlate with their intentionality ratings in Harm vignettes.

### Materials and Methods

One hundred and eighty participants were recruited on Amazon’s Mechanical Turk (AMT)^1^ and completed the survey for a monetary payment of $0.40. Participants were randomly assigned to one of our two experimental conditions, which correspond to the presentation of either the Harm or the Help version of the chairman vignette. Participants answered four questions that served as inclusion criteria: two Attention Check Questions (ACQs), a question about familiarity with the experimental task, and a question about the experimental hypothesis^2^. Of the original sample, eight participants failed the Attention Check Questions (ACQs), 27 participants affirmed being familiar with the chairman scenario, and four participants mentioned a relationship between our independent and dependent variables when asked about the experimental hypothesis. After excluding these participants, we had a final sample of 141 participants (58 males, 82 females, 1 other; *M*_age_ = 39.26 years, *SD* = 13.88 years, age range 20–71 years).

As a cover story, participants were informed they were going to participate in two separate studies, which had been pooled together for the sake of convenience. This is a common procedure used to avoid demand effects (Parrott and Hertel, 2005). The first study was introduced as a “Self-Evaluation Questionnaire” about feelings in daily life, and included the TAS along with the demographic questions. In the second study, labeled “Attitudes toward hypothetical situations,” participants read the chairman vignette from the SEE seminal study in its “harm” or “help” version (see Introduction in this paper). Afterward, participants answered two different questions about the scenario. First, participants rated how much they agree with the following statement “The chairman of the board intentionally harmed (helped) the environment” on a scale from 1 (“strongly disagree”) to 7 (“strongly agree”). Second, they responded whether the chairman of the board should be blamed (praised) for harming (helping) the environment on a scale from 1 (“definitely yes”) to 7 (“definitely no”).

### Results

First, we explored the data to see if parametric test assumptions were met. Normal Q–Q plots and Kolmogorov–Smirnov test showed that intentionality scores for Harm, *D*(61) = 0.308, *p* < 0.001, and Help, *D*(80) = 0.347, *p* < 0.001, both significantly deviated from normal. The same happened with responsibility scores for Harm, *D*(61) = 0.366, *p* < 0.001, and Help, *D*(80) = 0.256, *p* < 0.001. Thus, we used non-parametric tests to analyze our data: Mann–Witney for comparisons between conditions, and Spearman’s Rho for correlations.

Moral valence of the side-effect influenced intentionality judgments. Participants were more willing to view the chairman’s action as intentional when the side effect was harming the environment (*Mdn* = 7.00) than when the side-effect was helping the environment (*Mdn* = 1.00), *U* = 200, *z* = -9.66, *p* < 0.001, *r* = 0.81. Intentionality ratings in the Harm case were significantly related to blame, *r*_s_ = 0.675, *p* < 0.001, and the intentionality attributed in the Help case was significantly related to praise, *r*_s_ = 0.447, *p* < 0.001.

**FIGURE 1:**
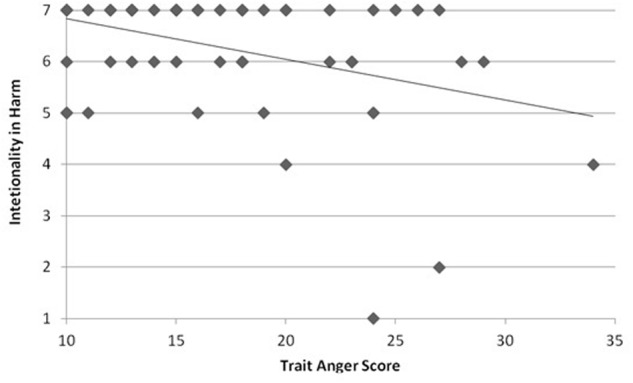
**Relationship between intentionality attribution in Harm case and TAS score**. Participants who scored higher in dispositional anger, as measured by Spielberg’s TAS, tended to rate the outcome of harming the environment as less intentional.

As the ABH states that affective reactions to the chairman action and its outcomes explain the higher intentionality ratings in Harm cases, but not in Help cases, we analyzed the relationship between TAS scores and intentionality ratings in Harm and Help separately. TAS scores were not significantly related to intentionality in Help cases, *r*_s_ = 0.150, *p* = 0.184. TAS scores were significantly related to intentionality in Harm cases, but negatively correlated, *r*_s_ = -0.273, *p* = 0.033 (**Figure 1**). Finally, blame ratings were not significantly related with TAS scores, *r*_s_ = -0.212, *p* = 0.102.

### Discussion

While we replicated the SEE, we failed to find support for the ABH: a higher disposition to get angry (as measured by the Trait Anger Scale) was not related with higher ratings of intentionality or blame in Harm cases. To the contrary, higher rankings in the TAS were associated with lower intentionality ratings. Considering the possibility that the Trait Anger Scale might not be a sensitive enough measure of the involvement of anger in these judgments, or the possibility that participants did not experience anger at all, we devised a second study in which we directly manipulated participants’ affective state using anger elicitation.

## Study 2: Anger Elicitation

Emotion elicitation methods are commonly used to test the role of affective processes in judgment and decision making (Harmon-Jones et al., 2007). In particular, we employed autobiographical recall, which is a widely used emotion elicitation technique (Lench et al., 2011; Quigley et al., 2014) and has been validated for internet-based designs (Mills and D’Mello, 2014; Ferrer et al., 2015). Writing about an emotional experience before the experimental task modifies participants’ affective state, and the influence of that affective state in judgment can be measured comparing to a control group.

The ABH posits that emotional reactions underlie the asymmetric attributions of intentionality between Harm and Help cases. Thus, it predicts that participants in a more intense emotional state (anger in particular) would show a more pronounced asymmetry in their intentionality judgments between Harm and Help cases, because they will show higher intentionality ratings in Harm cases.

### Materials and Methods

Three hundred and five participants were recruited on Amazon’s Mechanical Turk (AMT) and filled the survey in exchange for $0.80. Participants were randomly assigned either to the anger elicitation group or the control group. As in Study 2, we included two ACQs, a question about familiarity with the chairman vignette, and another question regarding suspicion about the experimental hypothesis. Twenty-one participants failed both ACQs, 27 were familiar with the chairman vignette, and 19 suspected a relationship between the emotion induction task and their responses to the chairman case, leaving a final sample of 238 participants (88 males, 150 females; *M*_age_ = 35.67 years, *SD* = 12.15 years, age range 18–71 years).

The same “separate studies” cover story of Study 1 was used to control for demand effects. The first study, labeled “memory for life events,” was the autobiographical recall emotion induction. Participants in the anger condition were instructed to write down the three things that make them most angry. Afterward, they were told to describe in detail one situation that made them feel extremely angry (Lerner and Keltner, 2001). They were instructed to provide as much detail as possible and to vividly recall what happened and how they felt (Strack et al., 1985). Participants in the control condition wrote about the last time they went grocery shopping, and listed the three things that they buy most often when they go grocery shopping. In the second study, they read both the Help and Harm versions of the chairman vignette, whose order of presentation was counterbalanced. Afterward, they rated on a scale from -10 (“completely disagree”) to +10 (“completely agree”) how much they agreed with the following statements: “The chairman of the board intentionally harmed (helped) the environment” and “The chairman of the board is accountable for harming (helping) the environment.” We chose a 21-point Likert scale to increase discriminating power (Preston and Colman, 2000).

To confirm that our emotion elicitation worked, we included a manipulation check in which participants self-reported their current feelings. They were instructed to rate on a 0 (“not at all”) to 8 (“extremely”) scale to what extent they were experiencing a list of different emotions. We included two anger-related words (“angry” and “irritated,” α = 0.874), and five more categories corresponding to other fundamental emotions: “fearful,” “happy,” “repulsed,” “sad,” and “surprised.” Because it has been shown that labeling emotions after the experimental manipulation can reduce their influence in subsequent judgments (Keltner et al., 1993b), emotional ratings were presented at the end of the survey.

### Results

First, we subtracted participants’ intentionality ratings in the Help scenario to their intentionality ratings in the Harm scenario to obtain an “asymmetry” measure. Normal Q–Q plots and Kolmogorov–Smirnov tests showed that scores for asymmetry, intentionality, accountability, and the different emotion categories in our manipulation check were not normally distributed across groups (all *p* < 0.05). Thus, we employed non-parametric tests to analyze our data: Mann–Witney *U* test for comparisons between conditions, and Spearman’s Rho for correlations.

We again replicated the SEE, as participants attributed more intentionality in Harm cases (*Mdn* = 21.00) than in Help cases (*Mdn* = 1.00), *z* = -12.45, *p* < 0.001, *r* = -0.81. There was a significant relationship between intentionality and accountability ratings, both for HARM, *r*_s_ = 0.620, *p* < 0.001, and Help cases, *r*_s_ = 0.273, *p* < 0.001.

Participants’ self-reported anger scores were significantly higher in the experimental group (*Mdn* = 1.00) than in the control group (*Mdn* = 0.00), *U* = 5067.00, *z* = -4.30, *p* < 0.001, *r* = -0.28. Although to a lesser degree, disgust ratings were also significantly higher in the experimental group (*Mdn* = 0.00), compared to the control group (*Mdn* = 0.00), *U* = 5844.00, z = -2.80, *p* = 0.005, *r* = -0.18. All ratings for other emotion categories did not significantly differ between conditions (all *p* > 0.05).

Asymmetry scores in the anger condition (*Mdn* = 16.00) did not differ significantly from control condition (*Mdn* = 17.00), *U* = 7030.00, *z* = -0.083, *p* = 0.934, *r* = -0.05. The SEE asymmetry was as large in the anger group (Harm *Mdn* = 21.00, Help *Mdn* = 1.00) as it was in the control group (Harm *Mdn* = 21.00, Help *Mdn* = 1.00). Accountability ratings were similar across conditions, both for Harm, *U* = 6878.50, *z* = -0.421, *p* = 0.674, *r* = -0.03, and Help cases, *U* = 65780.50, *z* = -0.938, *p* = 0.348, *r* = -0.06.

### Discussion

Again, our results failed to confirm the ABH predictions. The SEE was elicited, but while the anger induction procedure was effective, it had no effect on intentionality and accountability ratings. However, one could argue that these results are due to a ceiling effect, given that intentionality ratings were maximal. In Study 3 we introduced changes in our experimental design in order to address this problem.

## Study 3: Anger Elicitation and Mitigating Factors

To avoid the problem of ceiling effects, our third study used vignettes involving a “mitigating factor” (Seidel and Prinz, 2013). Previous research has shown that manipulating the valence of the agent’s main goal (from making profit to a generous one) reduces intentionality ascriptions in Harm cases (Shepherd, 2012). Thus, we used cases in which the valence of the side effect is negative, but the agent’s main goal is positive. As affective processes are supposed to play a role only in intentionality attributions in Harm cases, and in order to increase power, we did not include Help cases. We selected two scenarios from the previous literature: Planner (Phelan and Sarkissian, 2008) and Modified Lieutenant (Phelan and Sarkissian, 2009). They were simplified in order to control for possible influences of the different wordings (Ngo et al., 2015).

In addition to using scenarios involving a mitigating factor, we also introduced a new emotion condition: amusement. Research has shown that inducing amusement in participants can reduce responses which are associated with negative emotional reactions to the task at hand, such as “deontological responses” to the footbridge dilemma (Valdesolo and Desteno, 2006). As the ABH posits that affective reactions explain attributions of intentionality in Harm cases, much like affective reactions explain deontological responses in the footbridge dilemma, we predicted that participants in the amusement condition would attribute less intentionality in SEE scenarios involving harmful side-effects than those in the control condition.

### Materials and Methods

Two hundred and fifty-three participants were recruited on Amazon’s Mechanical Turk (AMT) and filled the survey in exchange for $0.70. Questions about familiarity, suspicion, and attention check questions were the same as in Study 2. Participants were randomly assigned to one of our three emotional manipulation conditions: anger, control or amusement. Eleven participants failed the two ACQs, 2 were familiar with the vignettes we used, and 25 were suspicious about the experimental hypothesis, leaving a final sample of 215 participants (86 males, 129 females; *M*_age_ = 34.43 years, *SD* = 11.25 years, age range 19–72 years).

The cover story and emotion induction were the same as in Study 2. In the new amusement condition, participants were told to list the three things that make them laugh the most, and then were instructed to write for 5 min about a personal experience that made them feel extremely amused. After the emotion elicitation, participants received both the following vignettes:

Planner (Phelan and Sarkissian, 2008):

The city planner started a plan to address the pollution problem.
He did not care about the effect the plan would have on joblessness.
He knew his plan would increase joblessness.

Modified Lieutenant (Phelan and Sarkissian, 2009):

The lieutenant sent a squad of soldiers to Thomson Hill in order to succeed in the campaign.
He did not care about the effect the strategy would have on soldiers.
He knew the soldiers sent to Thomson Hill would be in the line of fire and some of them would be killed.

Order of presentation was counterbalanced. The intentionality question was “Did the city planner/lieutenant intentionally raise joblessness levels/cause the soldiers’ death?” which participants responded in a 1 (“not intentional at all”) to 8 (“completely intentional”) scale. Participants rated whether the vignette’s protagonist should be blamed for his action’s side-effect from 1 (“not blameworthy at all”) to 8 (“completely blameworthy”).

The manipulation check was presented after the intentionality and responsibility judgments. It consisted of seven items: “angry,” “amused,” “fearful,” “repulsed,” “sad,” and “surprised.” Participants rated to what extent they were experiencing those feelings on a 0 (“not at all”) to 8 (“extremely”) scale.

### Results

Intentionality ratings for Planner and Lieutenant scenarios yielded relatively high reliability (α = 0.712). Thus, we created a composite score by averaging the intentionality ratings across the two scenarios, and used it as the dependent variable in our main analysis. We did the same with blame scores (α = 0.515). Once again, linear model assumptions were not met. Normal Q–Q plots and Kolmogorov–Smirnov test showed that scores for intentionality, blame, and emotion categories were not normally distributed (all *p* < 0.05). Thus, we used non-parametric tests to analyze the data: Kruskal–Wallis for comparisons between groups, and Spearman’s Rho for correlations.

Our manipulation significantly affected participants self-reported amusement, *H*(2) = 17.98, *p* < 0.001. Pairwise comparisons with adjusted *p*-values showed that there were significant differences in amusement between control and amusement groups (*p* = 0.005, *r* = 0.26), and between anger and amusement groups (*p* < 0.001, *r* = 0.35), but not between anger and control groups (*p* = 0.840, *r* = 0.09). Anger ratings were also affected by our manipulation, *H*(2) = 32.96, *p* < 0.001. There were significant differences in self-reported anger between control and anger groups (*p* < 0.001, *r* = 0.36), and between anger and amusement groups (*p* < 0.001, *r* = 0.47), but not between amusement and control groups (*p* = 0.547, *r* = 0.11). Our manipulation also affected participants’ levels of sadness, *H*(2) = 6.91, *p* = 0.032. There were significant differences in sadness between amusement and anger groups (*p* = 0.026, *r* = 0.23), although not between amusement and control groups (*p* = 0.362, *r* = 0.13), neither between anger and control groups (*p* = 0.702, *r* = 0.10). Other emotions were not affected by our manipulation (all *p* > 0.05).

**FIGURE 2:**
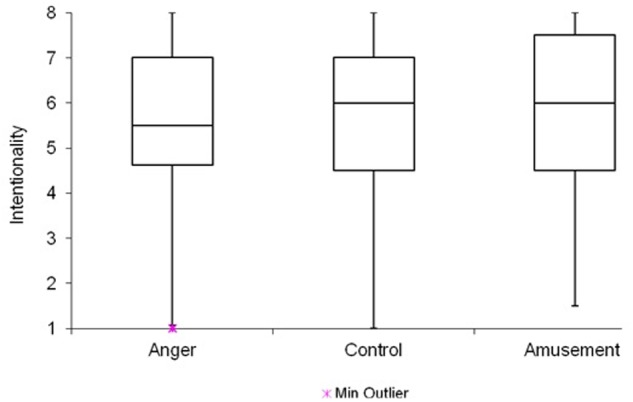
**Emotion condition by intentionality scores**. Despite addressing the possibility of ceiling effects, emotion manipulation had no effect.

Descriptive analysis showed that scores did not cluster at the end of the scale neither for intentionality (*Mdn* = 6.0) nor blame (*Mdn* = 6.0). Intentionality ratings were not significantly affected by emotion, *H*(2) = 1.26, *p* = 0.533 (**Figure 2**), and neither was blame, *H*(2) = 5.16, *p* = 0.076. Intentionality ratings were again significantly related to responsibility scores, *r*_s_ = 0.572, *p* < 0.001.

### Discussion

After controlling for ceiling effects, our third experiment failed again to find support for ABH. Although intentionality and blame ratings were not maximal, they remained unaffected by emotion. The results reinforce those obtained in Study 2 and add more evidence against the role of affective processes in the SEE.

## General Discussion

The motivational bias account for the SEE posits the possibility of affective reactions playing a role on intentionality attributions (ABH), because affective reactions can heighten negative evaluations of the situation at hand. These negative evaluations are supposed to trigger a motivation to blame, which in turn acts expansively on intentionality ascriptions. After reviewing the available empirical tests for the ABH and the literature about emotion and blame, we hypothesized that the affective reactions underlying the SEE could be confined to anger. In three different studies, we failed to find a relationship between anger and intentionality ratings in SEE cases.

In our first study, participants’ tendency to feel anger as measured by Spielberger’s TAS was in fact negatively correlated with intentionality ascriptions in SEE cases. In our second study, inducing anger in participants by making them write about a situation in which they felt intense anger did not affect their intentionality ratings, a result which was replicated in study 3 after addressing the problem regarding possible ceiling effects. Inconsistent with the existing literature on the topic, we did not find a relationship between anger and blame in any of our studies. However, we systematically found a relationship between responsibility judgments and intentionality judgments.

There are some limitations in our studies. Regarding Study 1, Spielberger’s TAS may not be the best choice when studying moral anger responses, and participants that scored higher in the TAS scale might not experience higher anger reactions to the chairman vignette. Our emotion elicitation technique has limitations too, since there were significant differences across groups in self-reports of emotions that were not the target of our manipulation. In study 2, our manipulation was successful in inducing anger on participants, but it also induced another negative emotion, disgust. In study 3, apart from anger and amusement, our manipulation influenced participants’ levels of reported sadness. However, other widely-used emotion elicitation methods suffer from the same problems. For example, film clips selected for inducing anger also elicit significant changes in participants’ levels of disgust and sadness (Gross and Levenson, 1995; Rottenberg et al., 2007). Furthermore, and of special interest here, anger-inducing film clips have been successfully used to increase participants blame attributions (Lerner et al., 1998; Goldberg et al., 1999).

Our results are in line with the conclusions of Young et al. (2006) and Cardinale et al. (2014). As we mentioned in the introduction, VMPFC patients and psychopaths are capable of angry responses, and thus these studies left open the possibility of anger playing a role in attributions of intentional action in SEE cases. However, our results suggest that anger does not play a role in the SEE. Our investigation completes these studies, answering this possible objection to them, and adds to the literature a new empirical study that speaks against the ABH.

On the other hand, the interpretation of our results seems inconsistent with those from Ngo et al. (2015), who claimed that the relationship they found between emotional reaction and intentionality ratings in SEE Harm cases supports the ABH. Ngo et al. (2015) interesting methodology notwithstanding, there are some limitations in their study that might be relevant here. First, since they do not systematically manipulate participants’ emotion, it is controversial to interpret their results in terms of causation. Second, their “emotional reaction” measure has limitations. Participants rated “How harming (or helping) the environment make you feel?” in a -3 (very negative) to 3 (very positive) scale. By stating the object, participants could have interpreted the question as asking for a (non-necessarily emotional) evaluation of the event (Alicke, 2008), or a “cold” emotional attitude instead of an emotional episode (Hacker, 2004). Third, the fact that this measure also correlated with brain activity in the amygdala in Harm cases is considered as evidence for the presence of emotion, as the amygdala has been related to emotion in several fMRI studies. However, inferring the presence of a particular cognitive process by finding activity in one particular brain region, or reverse inference (Poldrack, 2006), is unjustified because most brain regions have been related to several different cognitive processes (Miller, 2008). In particular, the amygdala has also been associated with processes, such as processing novel material, that can be emotional or non-emotional (Pessoa, 2010; Barret and Satpute, 2013).

## Conclusion

In this paper, we tested the role of anger on intentionality attributions for negative side effects to which the agent expresses indifference. The results across our three studies, together with those of Young et al. (2006) and Cardinale et al. (2014), are inconsistent with the ABH. It is important to note that our studies do not offer evidence against the motivational bias hypothesis (Nadelhoffer, 2006; Alicke and Rose, 2012) *per se*, but against this motivational bias being driven by anger. Although our data suggest that anger do not play a role on intentionality attributions in SEE scenarios, responsibility judgments consistently correlated with intentionality attributions in both Harm and Help cases across all of our studies. Thus, a motivation to blame the agent could be partially explaining intentionality ascriptions in SEE cases. This lack of relationship between anger and blame is perhaps our most surprising finding, taking into account the existing literature on the topic. Our second and third studies failed to find a relationship between anger and blame, and even more strikingly, our first study found a negative relationship between both.

We suggest that further studies should test the motivational bias hypothesis without considering it to be necessarily driven by affective processes, as accumulating evidence suggest this is not the case. On the other hand, we call for further testing and reassessment of the relationship between anger and blame.

## Ethics Statement

The project was approved by the research ethics committee of the University of the Balearic Islands (Comité d’ÈTica de la recerca de la Universitat de les Illes Balears).

## Author Contributions

RD, HV, and AG conceptualized and designed the experiments, and interpreted the results. RD acquired and analyzed the data.

## Conflict of Interest Statement

The authors declare that the research was conducted in the absence of any commercial or financial relationships that could be construed as a potential conflict of interest.

## Footnotes

1 Using AMT as a participant pool is a validated method for data collection in the social sciences, being that participants are more demographically diverse than standard Internet and college students samples, and producing data as reliable as that obtained using traditional pen-and-paper methods (Buhrmester et al., 2011).

2 ACQs include their correct answer on the very wording of the question (Oppenheimer et al., 2009). Thus, incorrect responses show that the participant is not paying enough attention to the instructions. As research suggest that participants familiarity with the experimental task could also be an important problem in crowdsourcing services such as AMT (Chandler et al., 2014), and can reduce effect sizes (Chandler et al., 2015), excluding participants that are familiar with the experimental task further ensures the quality of the data. Finally, asking participants about the experimental hypothesis allows us to control for demand effects.
